# Genetic parameter analysis of reproductive traits in Large White pigs

**DOI:** 10.5713/ab.22.0119

**Published:** 2022-09-02

**Authors:** Guanghui Yu, Chuduan Wang, Yuan Wang

**Affiliations:** 1 College of Animal Science and Technology, Qingdao Agricultural University, Qingdao 266109, China; 2 College of Animal Science and Technology, China Agricultural University, Beijing 100193, China

**Keywords:** Genetic Parameter, Pig, Reproductive Trait

## Abstract

**Objective:**

The primary objective of this study was to determine the genetic parameters for reproductive traits among Large White pigs, including the following traits: total number born (TNB), number born alive (NBA), litter birth weight (LBW), average birth weight (ABW), gestation length (GL), age at first service (AFS) and age at first farrowing (AFF).

**Methods:**

The dataset consisted of 19,036 reproductive records from 4,986 sows, and a multi-trait animal model was used to estimate genetic variance components of seven reproductive traits.

**Results:**

The heritability estimates for these reproductive traits ranged from 0.09 to 0.26, with the highest heritability for GL and AFF, and the lowest heritability for NBA. The repeatabilities for TNB, NBA, LWB, ABW, and GL were ranged from 0.16 to 0.34. Genetic and phenotypic correlations ranged from −0.41 to 0.99, and −0.34 to 0.98, respectively. In particular, the correlations between TNB, NBA and LBW, between AFS and AFF, exhibited a strong positive correlation. Furthermore, for TNB, NBA, LBW, ABW, and GL, genetic correlations of the same trait between different parities were moderately to strongly correlated (0.32 to 0.97), and the correlations of adjacent parities were higher than those of nonadjacent parities.

**Conclusion:**

All the results in the present study can be used as a basis for the genetic assessment of the target population. In the formulation of dam line selection index, AFS or AFF can be considered to combine with TNB in a multiple trait swine breeding value estimation system. Moreover, breeders are encouraged to increase the proportion of sows at parity 3–5 and reinforce the management of sows at parity 1 and parity ≥8.

## INTRODUCTION

In the pig industry, female reproductive traits are the most functional traits affecting the economic benefit of pig production. According to previous studies, reproductive problems cause approximately 30% of culling in pig production systems [1]. Thus, increasing attention has been given for reproductive performance. Recently, advanced breeding technologies based on the best linear unbiased prediction have been applied to the breeding process of animal economic traits [2,3]. Understanding genetic parameters of these trait is required to accurately estimate breeding values, which is helpful for the formulation of selection indices and breeding schemes in pig production, and it is worth mentioning that reproductive traits account for a large proportion of the establishment of dam line selection indices by different population [4,5]. Meanwhile, genetic parameters are population specific, and it is essential to estimate the genetic parameters of different populations [6].

To date, many studies have been performed to estimate the genetic parameters of economic traits in pigs, such as growth trait [7], semen traits [8], and litter size traits [9]. These results demonstrate the importance of genetic evaluation of economic traits in improving productivity. However, most sows show different physiological development at different parities, which may influence the reproductive performance [10]. Therefore, obtaining phenotypic records at different parities is important to accurately estimate genetic parameters. Besides, in previous studies, some researchers considered different parities as a single trait [2,11], while others considered that a partially different genetic control of prolificacy between parity 1 and later parities, and treated the first and later parities as different traits [12,13]. Thus, understanding the genetic correlation between different parities is also helpful for the rational division of traits in the process of genetic parameter estimation.

Based on the above studies, the purpose of this study was to estimate the genetic parameters of female reproductive traits in Large White pigs, including total number born (TNB), number born alive (NBA), litter birth weight (LBW), average birth weight (ABW), gestation length (GL), age at first service (AFS), and age at first farrowing (AFF). The results of this study will facilitate the development of a genetic evaluation system for female reproductive traits, and improve the reproduction efficiency of pigs.

## MATERIALS AND METHODS

### Ethics statement

All experiments involved in this study followed the “Guidelines for Experimental Animals” of the Ministry of Science and Technology (Beijing, China). Operations and animal care were sustained by the experimental animal ethics committee of China Agricultural University (approval number: GB/T 17236-2008).

### Data

The population used for this study is from the nucleus pig breeding farm of Beijing Shunxin Agriculture Co., Ltd., Beijing, China. The core group of this farm is comprised approximately 600 sows and 50 boars. These individuals are fed and managed in a consistent manner. The sows were selected based on the dam line selection index, which was composed of the traits with TNB, backfat at 100 kg live weight, and age at 100 kg live weight. The selection intensity of breeding sows is 12% and the replacement rate is about 56%. Furthermore, culling is usually passive, mainly caused by individuals with genetic defects or disease.

A total of 19,306 farrowing records, generated from 570 boars and 4,986 sows, were collected from 2007 to 2016. Seven reproductive traits (i.e., TNB, NBA, LBW, ABW, GL, AFS, and AFF) were considered in this study. TNB was defined as the total number of piglets born per litter, NBA was defined as the number of piglets born alive, LBW was defined as the total weight of alive piglets, ABW was defined as the average weight of each individual, GL was defined as the interval between insemination and farrowing, AFS was defined as the interval from birth to first insemination and AFF was defined as the interval from birth to first farrowing. According to the data distribution and prior knowledge, TNB, NBA, LBW, ABW, and GL were approximately normally distributed, but for AFS and AFF, we used the rntransfrom function in the GENABEL R package to normalize the phenotype values. Data within the mean plus or minus three standard deviations were retained in the present study.

### Statistical analysis

In this study, we first analyzed the effects of herds, years, seasons, and parities on reproductive traits using PROC general linear model in SAS 9.2 (SAS Institute Inc., Cary, NC, USA). The linear model was used as Model A:


$$\text{Model A:}\;{\text{Y}}_{\text{ijkl}}=\mu +{\text{h}}_{\text{i}}+{\text{m}}_{\text{j}}+{\text{s}}_{\text{k}}+{\text{p}}_{\text{l}}+{\text{e}}_{\text{ijkl}}$$

where Y_ijkl_ is the phenotypic value of each trait; μ is the overall mean; h_i_ represents the fixed effect of farms, which contains 2 herds; m_j_ represents the fixed effect of farrowing year, which includes 10 levels (from 2007 to 2016) ; s_k_ represents the fixed effect of season, which includes 4 seasons (spring: form March to May; summer: from June to August; autumn: from September to November; winter: from December to February); pl represents the fixed effect of parity, which includes 8 levels (1, 2, 3, 4, 5, 6, 7, and ≥8); e_ijkl_ is the random residual corresponding to the trait observation value. Significance was tested using Duncan's multiple comparison test.

A multi-trait animal model was used to estimate the genetic variance components. The model B was fitted for TNB, NBA, LBW, ABW, and GL, model C was fitted for AFS and AFF:


$$\begin{array}{l} \text{Model B:}\;Y=X\beta +\mathrm{Za}+\mathrm{Wpe}+e\\ \text{Model C:}\;Y=X\beta +\mathrm{Za}+e \end{array}$$

where in Models B and C, **Y** is the vector of observation; **β** is the vector of fixed effects (in Model B, fixed effects contain herd, year, season and parity; in Model C, fixed effects contain herd, year and season); **a** is the vector of additive genetic effects; **pe** is the vector of permanent environments of individuals for TNB, NBA, LBW, ABW, and GL; **e** is a vector of residuals, and **X**, **Z**, and **W** are incidence matrices associated with **β**, **a**, and **pe**, respectively. In addition, for TNB, NBA, LBW, ABW, and GL, we calculated the genetic correlation and phenotypic correlation between the same reproductive trait at different parities using Model C.

In this study, we used a restricted maximum likelihood procedure to estimate the variance components of each trait in ASReml software [14]. Heritabilities, repeatabilities, genetic correlations and the proportion of phenotypic variance explained by permanent environmental effects were calculated as follows:


$$\begin{array}{l} h^{2}=\frac{\sigma_{a}^{2}}{\sigma_{p}^{2}},\;\;\;re=\frac{\sigma_{\text{a}}^{2}+\sigma_{pe}^{2}}{\sigma_{\text{p}}^{2}};\\ r_{\text{A}}=\frac{cov(a_{1},a_{2})}{\sigma_{a1}\sigma_{a2}};\;\;\;pe^{2}=\frac{\sigma_{pe}^{2}}{\sigma_{p}^{2}} \end{array}$$

where *h*^2^ is the heritability; $\sigma_{a}^{2}$ is the additive genetic variance; $\sigma_{pe}^{2}$ is the permanent environmental variance; $\sigma_{p}^{2}$ is the phenotypic variance; re is the repeatability for TNB, NBA, LBW, ABW and GL; *r*_A_ is the genetic correlation between trait 1 and trait 2, *cov*(*a*_1_, *a*_2_)is the genetic covariance of two traits; $\sigma_{a1}^{2}$ and $\sigma_{a2}^{2}$ refer to the genetic standard deviation of trait 1 and trait 2, respectively; and *pe^2^* is the proportion of phenotypic variance caused by permanent environmental effects. In addition, standard errors (SEs) of heritability were calculated using the formula given by Klei and Tsuruta [15]. Genetic correlations and phenotypic correlations were calculated by ASRmel software.

## RESULTS

### Phenotypic statistics

Descriptive statistics of the seven reproductive traits are shown in Table 1. The number of records of TNB, NBA, LBW, ABW, and GL were nearly consistent, while AFS and AFF were relatively small, mainly because these two traits are only from the reproductive records of parity 1. Except for GL, the coefficients of variation of the other traits were greater than 10%, which indicated that these traits can provide some progress by developing reasonable breeding plans.

### Fixed effects analysis

As shown in Supplementary Table S1, the effect of years and parities were extremely significantly (p<0.01) associated with seven reproductive traits, while the effect of herds were extremely significantly (p<0.01) associated with TNB, ABW, GL, AFS, and AFF, the effect of seasons were significantly (p<0.01) or extremely significantly (p<0.01) associated with TNB, ABW, AFS, and AFF. Therefore, different factors should be considered in the model of genetic variance component estimation. In addition, the results of multiple comparison test were shown in Supplementary Table S2–S5. Among them, Supplementary Table S4 showed the effect of years on reproductive traits, and the best performance was in 2015 and 2016. Supplementary Table S5 showed the effect of parities on reproductive traits, and the reproductive performance of sows was best at parity 3–5, while the performance at parity 1 and parity ≥8 were worse than other parity.

### Genetic parameter analysis

The estimates of genetic variance components and genetic parameters were shown are Table 2. Among them, additive genetic variances ranged from 0.005 (ABW) to 0.858 (GL), and the proportion of phenotypic variance caused by permanent environmental effects was lower than 0.1. The heritabilities of the 7 reproductive traits varied from 0.09 to 0.26, with the highest heritability for GL and AFF, and the lowest heritability for NBA. The repeatabilities for TNB, NBA, LWB, ABW, and GL were ranged from 0.16 to 0.34.

As shown in Table 3, genetic and phenotypic correlations between different traits were ranged from −0.41 to 0.99, and −0.34 to 0.98, respectively. In particular, estimated genetic correlations between TNB, NBA and LBW, and between AFS and AFF, were larger than 0.8, which showed a strongly positive correlation. In the process of breeding, the performance of several other traits can be improved by selecting a certain trait, while the genetic correlations between ABW and TNB, NBA exhibited a moderate negative correlation. With the increase in the number of piglets born, the individual birth weight gradually decreased, which also led to an increasing number of weak piglets. Phenotypic correlations, such as between TNB, NBA, and LBW, showed a trend consistent with genetic correlations. Furthermore, for TNB, NBA, LBW, ABW, and GL, the genetic correlations of one trait in different parities were moderately to strongly correlated (0.32 to 0.97), and the correlations of adjacent parities were higher than those of nonadjacent parities (Table 4). However, the phenotypic correlations of one trait in different parities ranged from 0.10 to 0.38, which showed a weak to moderate correlation.

## DISCUSSION

Understanding the genetic parameters of the target population is crucial for making animal breeding programs, which helps to predict the response to selection and monitor genetic progress [6]. Seven economically important traits related to sow efficiency (i.e., TNB, NBA, LBW, ABW, GL, AFS, and AFF) were considered in this study, and the results, such as the influence of environmental factors, the estimates of heritabilities and genetic correlations between different traits, can be used to provide useful information for the further breeding process of the population. Currently, only TNB trait is considered in the dam-line selection index recommended by the China Swine Genetic Improvement Program, more reproductive traits should also be taken into account [9]. Thus, knowledge of genetic correlations of TNB with other economically important prolificacy traits is required for combining these traits in a multiple trait swine breeding value estimation system.

As we known, both environmental and genetic factors can affect the reproductive performance of sows. We analyzed the effects of herd, year, season, and parity on reproductive traits in this study. Results showed that different factors need to be considered when making mating plans. Among them, breeders paid more attention to the effect of parities. Some researchers found that the sows have better reproductive performance at or beyond third parity [11,16]; however, Takai and Koketsu [17] considered that the best performance of sows was at the first or second parity. In our study, the best performance was at parity 3–5, while parity 1 and parity ≥8 were worse than other parity. Thus, breeders should implement different management and feed design for different parities. For instance, sows at first parity need more than 10% to 15% protein to sustain their own growth, while sows at higher parity require more energy for maintenance [11,18]. Meanwhile, the proportion of sows at parity 3–5 should also be increased to improve economic efficiency.

A multi-trait animal model was used to estimate the variance components of seven reproductive traits in this population. Among them, the estimates of the additive genetic variances were able to help animal breeders measure the genetic variations and determine the response to selection. The estimate of additive genetic variance for TNB was larger than those reported by Zhang et al [9], who estimated additive genetic variances ranging from 0.534 to 0.770 in the connected groups of Large White pigs, and Ye et al [11], who estimated the value of 0.786 in Large White pigs, while smaller than the results obtained by Thekkoot et al [19] and Zhang et al [2], who reported estimated additive genetic variances of 1.529 and 1.480, respectively. The estimate for NBA was similar to TNB, but the difference was that the additive genetic variance for NBA was smaller than that found by Ye et al [11] with a value of 0.786. In addition, the estimate of AFF was less than that from Zhang et al [9], while the estimate for GL was greater than that from Zhang et al [2]. The estimated variance components from different populations for the same traits are usually very close or fluctuate in a small range, and the differences between the estimation of variance components are mainly related to the population size and the dataset [20].

In this study, the estimated heritabilities of seven reproductive traits ranged from 0.09 to 0.26. Heritabilities for TNB and NBA were in the range reported in previous studies, for instance, Wolf et al [21] estimated heritabilities of 0.13 for TNB and 0.14 for NBA in a Czech Large White pig population, Zhang et al [2] estimated that the heritabilities of both traits were 0.13, and Hollema et al [22] estimated that the heritability for NBA was 0.11, which were slightly higher than our estimated heritabilities. Furthermore, our estimates of heritabilities were larger than those from Ye et al [11], who reported estimated heritabilities for these two traits of 0.07 and 0.06, respectively. Heritability for LBW was also consistent with published literature, such as the results of Wolf et al [21] and Ye et al [11], who reported estimated heritabilities of 0.06 and 0.13, respectively. The estimate of heritability for ABW was smaller than those from Kaufmann et al [23] and Hollema et al [22], with the estimated heritabilities of 0.21 and 0.29, respectively. The reason for this situation may be related to the breeding plan of the target population, which influences the piglet birth weight. In addition, the estimated heritabilities for GL, AFS and AFF were approximately 0.25. For GL, there were some differences among different populations. Zhang et al [2] and Hollema et al [22] estimated heritabilities of 0.14 and 0.21, respectively, which were slightly lower than the results in our study; and the heritability estimated by Hanenberg et al [24] was consistent with this study. However, due to the small phenotypic variation in GL, the possibility of improving the reproductive performance of sows by changing the gestation cycle was also reduced. For AFS and AFF, these two traits have received increasing attention because they can be treated as an evaluation of early reproductive performance of sows. The heritabilities of AFS and AFF were in the range of those obtained in previous studies, which ranged from 0.11 to 0.31 [9,24,25]. Only the first litter records can be used in estimating the heritabilities of AFS and AFF, and thus, the accuracy of the results can be improved by increasing the amount of data collected. Besides, the estimated repeatabilities for TNB, NBA, and LBW were consistent with the report of Zhang et al [9] and Chen et al [20], who reported the repeatabilities ranged from 0.14 to 0.17; while Lopez and Seo [13] estimated the repeatabilities of TNB and NBA were 0.07 and 0.06, respectively, lower than our results.

In the present study, genetic correlations between different traits ranged from −0.41 to 0.98. Among them, the estimated genetic correlations between TNB and NBA agreed with the results from Roehe et al [12], Zhang et al [9], and Ye et al [11], who reported genetic correlations between 0.87 and 0.98. Estimates of genetic correlation between TNB and NBA were high, which suggests that selection for either TNB or NBA will result in a relevant response on either of the traits not selected for. The genetic correlations between LBW and litter size traits were generally high and consistent with the results obtained by Wolf et al [21] and Schneider et al [26]. Therefore, in the established dam line selection index, selection based on TNB will improve NBA and LBW simultaneously. The genetic correlation between ABW and NBA was similar to the result estimated by Hollema et al [22], who estimated the parameter to be −0.4. Moreover, piglet birth weight can be used as a selection criterion to improve the survival of pre-weaned piglets, and occupy an important part of the selection index. However, the genetic correlations between ABW and GL showed a moderately positive correlation, which was inconsistent with the estimated genetic correlation that was close to 0 obtained by Hollema et al [22]. For AFS and AFF, there was a high positive genetic correlation between the two traits. AFF can be considered as a comprehensive trait that combines AFS and GL, and the variation in GL was small, which was an important factor leading to the strong positive correlation between them. This is consistent with the result obtained by Holm et al [27], who suggested that the portion that explains AFS genetic variation accounts for a large proportion of AFF genetic variation. Negative genetic correlations of AFS and AFF with litter traits indicated that sows with reduced AFS and AFF were observed to have increased values for the traits TNB and NBA [9]. Thus, AFS or AFF can be considered in the formulation of dam line selection index in the future. In addition, the phenotypic correlations showed consistent changes with the genetic correlations, which was similar to previous studies [9,11,22].

Genetic correlations between different parities for all traits were moderately to strongly positive. For TNB and NBA, genetic correlations between different parities ranged from 0.71 to 0.98, which was consistent with Ye et al [11] and Lopez and Seo [13], who reported that the genetic correlations varied from 0.48 to 0.99. In addition, genetic correlation between parity 1 and other parities showed a strong positive correlation (0.71 to 0.98) in our study, which was higher than those reported in the above research (0.48 to 0.74). Therefore, it is reasonable for us to consider different parities as the same trait. While the estimated phenotypic correlations between different parities were weaker than their corresponding genetic correlations, which also indicated that it was inadvisable to select sows based on the reproductive performance after only one parity. However, for other reproductive traits, there have been limited reports of correlations between different parities, our results can serve as an important reference.

## CONCLUSION

In this study, we estimated the genetic parameters for seven reproductive traits in a Large White pig population, which can be used as a basis for the genetic assessment of the target population, and improve the efficiency of breeding work. The performance of some traits, such as TNB, NBA, and LBW, showing a high genetic correlation, can be improved by selecting a certain trait. Meanwhile, AFS or AFF can also be considered in the formulation of dam line selection index in the future. In addition, increasing the proportion of sows at parity 3 to 5, and reinforcing the management of sows at parity 1 and parity ≥8 can be an effective method to improve economic efficiency.

## Figures and Tables

**Table 1:** Descriptive statistics of seven reproductive traits

Traits	Number	Unit	Average	SD	Min	Max	CV (%)
TNB	18,883	each	10.46	2.87	3	18	27.44
NBA	18,713	each	10.05	2.77	3	17	27.56
LBW	17,484	kg	14.21	3.92	2.66	29.2	27.59
ABW	17,484	kg	1.43	0.21	0.8	2.7	14.69
GL	18,982	day	115.1	1.81	105	127	1.57
AFS	4,362	day	282.38	51.13	148	440	18.11
AFF	4,362	day	397.82	51.25	260	557	12.88

TNB, total number born; NBA, number born alive; LBW, litter birth weight; ABW, average birth weight; GL, gestation length; AFS, age at first service; AFF, age at first farrowing; SD, standard deviation; Min, minimum; Max, maximum; CV, coefficient of variation.

**Table 2:** Estimates of variance components and genetic parameters for reproductive traits

Traits	$\sigma_{a}^{2}$	$\sigma_{pe}^{2}$	$\sigma_{e}^{2}$	$\sigma_{p}^{2}$	*pe* *^2^*	*h*^2^ (SE)	re
TNB	0.844	0.572	6.726	8.142	0.070	0.10(0.012)	0.17
NBA	0.692	0.538	6.355	7.585	0.071	0.09(0.012)	0.16
LBW	1.440	1.037	12.697	15.174	0.068	0.10(0.012)	0.16
ABW	0.005	0.004	0.033	0.042	0.095	0.12(0.014)	0.21
GL	0.858	0.265	2.184	3.307	0.080	0.26(0.019)	0.34
AFS	0.252	-	0.737	0.989	-	0.25(0.03)	-
AFF	0.260	-	0.726	0.986	-	0.26 (0.03)	-

$\sigma_{a}^{2}$ additive variance; $\sigma_{pe}^{2}$ , permanent environmental variance; $\sigma_{e}^{2}$, residual errors variance; $\sigma_{p}^{2}$, phenotypic variance; *pe^2^*, proportion of phenotypic variance explained by permanent environmental effects; *h*^2^, heritability, SE, standard error; re, repeatability; TNB, total number born; NBA, number born alive; LBW, litter birth weight; ABW, average birth weight; GL, gestation length; AFS, age at first service; AFF, age at first farrowing.

**Table 3:** Genetic correlation and phenotypic correlation between reproductive traits

Traits	TNB	NBA	LBW	ABW	GL	AFS	AFF
TNB	-	0.98 (0.005)^1)^	0.83(0.031) ^2)^	−0.39(0.075)	−0.26(0.069)	−0.11(0.09)	−0.11(0.09)
NBA	0.94(0.001)	-	0.83(0.029)	−0.41(0.079)	−0.21(0.072)	−0.13(0.09)	−0.12(0.09)
LBW	0.80(0.003)	0.87(0.002)	-	0.15(0.09)	−0.11(0.075)	−0.06(0.05)	−0.08(0.06)
ABW	−0.34(0.008)	−0.31(0.007)	0.15(0.008)	-	0.21(0.071)	0.04(0.08)	0.02(0.08)
GL	−0.15(0.009)	−0.13(0.009)	−0.08(0.009)	0.13 (0.01)	-	0.07(0.08)	0.15(0.07)
AFS	0.03 (0.015)	0.02 (0.016)	0.13(0.014)	0.051(0.02)	0.05(0.016)	-	0.99(0.01)
AFF	0.03 (0.013)	0.022(0.016)	0.13 (0.014)	0.05(0.018)	0.31(0.016)	0.98 (0.008)	-

TNB, total number born; NBA, number born alive; LBW, litter birth weight; ABW, average birth weight; GL, gestation length; AFS, age at first service; AFF, age at first farrowing.

1) The estimation of genetic correlations are shown in above diagonal, and phenotypic correlations are shown in below diagonal.

2) The estimated standard errors are shown in parentheses.

**Table 4:** Genetic correlation and phenotypic correlation of one trait at different parities

Traits	Parity			

	1	2	3	4
TNB				
1	-	0.91^1)^ (0.08)	0.77(0.126)^2)^	0.72(0.135)
2	0.18 (0.017)	-	0.98(0.09)	0.78(0.136)
3	0.12 (0.019)	0.20(0.019)	-	0.84(0.14)
4	0.16 (0.021)	0.20(0.021)	0.20(0.021)	-
NBA				
1	-	0.91(0.09)	0.71(0.15)	0.71(0.18)
2	0.16(0.017)	-	0.97(0.11)	0.88(0.17)
3	0.11(0.019)	0.19(0.019)	-	0.79(0.19)
4	0.14(0.021)	0.18(0.021)	0.19(0.021)	-
LBW				
1	-	0.92(0.1)	0.66(0.17)	0.32(0.22)
2	0.20(0.018)	-	0.93(0.16)	0.83(0.2)
3	0.11(0.02)	0.18(0.019)	-	0.78(0.25)
4	0.10(0.022)	0.18(0.022)	0.19(0.022)	-
ABW				
1	-	0.83(0.09)	0.72(0.11)	0.58(0.14)
2	0.23(0.017)	-	0.87(0.07)	0.66(0.14)
3	0.21(0.019)	0.31(0.023)	-	0.98(0.1)
4	0.18(0.022)	0.22(0.022)	0.28(0.021)	-
GL				
1	-	0.94(0.04)	0.76(0.06)	0.65(0.07)
2	0.32(0.016)	-	0.86(0.06)	0.85(0.05)
3	0.28(0.018)	0.34(0.018)	-	0.99(0.035)
4	0.27(0.021)	0.36(0.02)	0.38(0.019)	-

TNB, total number born; NBA, number born alive; LBW, litter birth weight; ABW, average birth weight; GL, gestation length.

1) The estimation of genetic correlations are shown in above diagonal, and phenotypic correlations are shown in below diagonal.

2) The estimated standard errors are shown in parentheses.

## SUPPLEMENTARY MATERIAL

**Supplementary Table S1**. The effect of different factors on reproductive performance traits **Supplementary Table S2**. The effect of herds on reproductive performance traits **Supplementary Table S3**. The effect of years on reproductive performance traits **Supplementary Table S4**. The effect of seasons on reproductive performance traits **Supplementary Table S5**. The effect of parities on reproductive performance traits
