# Supplementary material for: Genetic parameter analysis of reproductive traits in Large White pigs
Source: Anim Biosci. 2022 Sep 2;35(11):1649–55. doi: 10.5713/ab.22.0119 (PMC9659455; doi:10.5713/ab.22.0119)
Supplement: Supplementary file 1 [file ab-22-0119-suppl.pdf]

Table S1. The effect of different factors on reproductive performance traits

Traits	Herd		Year		Season		Parity	
	Df	F[1)]	Df	F	Df	F	Df	F
TNB	1	16.84**	9	33.97**	3	3.73*	7	49.02**
NBA	1	0.07	9	22.06**	3	2.27	7	43.98**
LBW	1	94.34**	9	29.26**	3	3.21*	7	129.59**
ABW	1	558.97**	9	53.12**	3	15.02**	7	114.36**
GL	1	121.23**	9	74.29**	3	1.15	7	31.24**
AFS	1	30.15**	9	21.29**	3	21.39**		
AFF	1	34.66**	9	21.14**	3	22.32**		

TNB, total number born; NBA, number born alive; LBW, litter birth weight; ABW, average birth weight; GL, gestation length; AFS, age at first service; AFF, age at first farrowing; Df, degree of freedom.

[1)] The effects of herds, years, seasons, and parities on reproductive traits. * $p<0.05$, ** $p<0.01$.

Table S2. The effect of herds on reproductive performance traits

Herd	TNB (each)[1)]	NBA (each)	LBW (kg)	ABW (kg)	GL (day)	AFS (day)	AFF (day)
1	10.31±2.96[2)]	9.98±2.94	14.61±4.24	1.48±0.23	114.95±1.74	277.81±49.43	392.75±49.6
	(8250, 43.69%)[a]	(8181, 43.72%)[3)]	(7669,43.86%)[a4)]	(7669, 43.86%)[a]	(8290, 43.67%)[a]	(1717, 39.36%)[a]	(1717,39.36%)[a]
2	10.56±2.80	10.11±2.62	14.13±3.75	1.40±0.19	115.21±1.86	285.3451.99	401.08±52.04
	(10633, 56.31%) [b]	(1053, 56.28%)	(9815, 56.14%)[b]	(9815, 56.14%)[b]	(10692, 56.33%)[b]	(2645, 60.64%)[b]	(2645, 60.64%))[b]

TNB, total number born; NBA, number born alive; LBW, litter birth weight; ABW, average birth weight; GL, gestation length; AFS, age at first service; AFF, age at first farrowing.

[1)] The unit of traits are shown in parentheses.

[2)] Phenotypic data of different herds are displayed by mean±standard deviation;

[3)]The number of individuals and frequencies in different herds are shown in parentheses, respectively;

[4)]Different superscript letters (a, b) in the same column mean significant differences (P<0.05).

Table S3. The effect of years on reproductive performance traits

Year	TNB (each)[1)]	NBA (each)	LBW (kg)	ABW (kg)	GL (day)	AFS (day)	AFF (day)
2007	10.44±3.19[2)] (2133, 11.30%)[a]	9.97±3.08 (2119, 11.32%)[3) a]	14.52±4.82 (1926, 11.02%)[ab4)]	1.46±0.24 (1926, 11.02%)[a]	115.33±1.91 (2119, 11.16%)[abc]	285.53±51 (791, 18.13%)[acd]	400.94±51.13 (791, 18.13%)[acd]
2008	10.19±2.89 (1494, 7.91%)[b]	9.85±2.84 (1485, 7.94%)[ab]	14.82±4.29 (1395, 7.98%)[c]	1.51±0.18 (1395, 7.98%)[b]	115.03±1.78 (1504, 7.92%)[d]	292.61±54.47 (319, 7.31%)[a]	407.62±54.6 (319, 7.31%)[a]
2009	10.33±3.00 (1723, 9.12%)[ab]	9.93±2.9 (1705, 9.11%)[ab]	14.37±4.05 (1609, 9.20%)[abd]	1.47±0.24 (1609, 9.20%)[ac]	115.26±1.72 (1741, 9.17%)[ab]	301.51±57.05 (235, 5.39%)[b]	416.63±57.4 (235, 5.39%)[b]
2010	10.35±2.95 (1839, 9.74%)[ab]	10.02±2.9 (1823, 9.74%)[a]	14.16±4.05 (1676, 9.59%)[d]	1.42±0.24 (1676, 9.59%)[d]	115.09±1.86 (1853, 9.76%)[d]	291.85±52.46 (323, 7.40%)[a]	407.28±52.55 (323, 7.40%)[a]
2011	10.08±3.01 (1552, 8.22%)[b]	9.77±3 (1541, 8.23%)[b]	13.89±4.15 (1421, 8.13%)[e]	1.44±0.24 (1421, 8.13%)[e]	115.42±1.78 (1564, 8.24%)[c]	279.08±51.63 (347, 7.96%)[d]	394.53±51.74 (347, 7.96%)[d]
2012	9.82±2.88 (1892, 10.02%)[c]	9.5±2.85 (1874, 10.01%)[c]	13.37±3.92 (1732, 9.91%)[f]	1.42±0.2 (1732, 9.91%)[d]	115.34±1.67 (1911, 10.07%)[abc]	289.12±56.46 (451, 10.34%)[ac]	404.77±56.66 (451, 10.34%)[ac]
2013	10.32±2.83 (1994, 10.56%)[ab]	10.03±2.78 (1960, 10.47%)[a]	14.18±3.89 (1888, 10.80%)[d]	1.42±0.2 (1888, 10.80%)[d]	115.37±1.89 (2007, 10.57%)[bc]	269.21±45.97 (496, 11.37%)[e]	385.29±45.95 (496, 11.37%)[e]

2014	10.68±2.43<br>(2472, 13.09%)^{d}	10.3±2.3<br>(2447, 13.08%)^{d}	14.26±3.35<br>(2374, 13.58%)^{ad}	1.39±0.16<br>(2374, 13.58%)^{f}	115.22±1.72<br>(2481, 13.07%)^{a}	282.37±50.77<br>(707, 16.21%)^{cd}	398.14±50.89<br>(707, 16.21%)^{cd}
2015	11.20±2.73<br>(2136, 11.31%)^{e}	10.58±2.53<br>(2125, 11.36%)^{e}	14.59±3.4<br>(2072, 11.85%)^{bc}	1.39±0.17<br>(2072, 11.85%)^{f}	114.51±1.74<br>(2146, 11.31%)^{e}	279.05±40.93<br>(341, 7.82%)^{d}	394.01±41<br>(341, 7.82%)^{d}
2016	10.94±2.59<br>(1648, 8.73%)^{f}	10.35±2.4<br>(1634, 8.73%)^{d}	15.43±3.63<br>(1391, 7.96%)^{g}	1.47±0.22<br>(1391, 7.96%)^{c}	114.361.68<br>(1656, 8.72%)^{f}	260.9438.75<br>(352, 8.07%)^{f}	375.55±38.89<br>(352, 8.07%)^{f}

TNB, total number born; NBA, number born alive; LBW, litter birth weight; ABW, average birth weight; GL, gestation length; AFS, age at first service; AFF, age at first farrowing.

1) The unit of traits are shown in parentheses.

2) Phenotypic data of different years are displayed by mean±standard deviation;

3)The number of individuals and frequencies in different years are shown in parentheses, respectively;

4)Different superscript letters (a, b, c, d, e, f, g) in the same column mean significant differences (P<0.05).

Table S4. The effect of seasons on reproductive performance traits

Season	TNB (each)[1)]	NBA (each)	LBW (kg)	ABW (kg)	GL (day)	AFS (day)	AFF (day)
Spring	10.54±2.84 (4684, 24.81%)^{a}[2)]	10.11±2.76 (4640, 24.80%)[3)]	14.43±3.96 (4371, 25.00%)^{a}[4)]	1.43±0.21 (4371, 25.00%)^{a}	115.09±1.78 (4692, 24.72%)	283.67±51.54 (1164, 26.69%)^{a}	399.12±51.76 (1164, 26.69%)^{a}
Summer	10.37±2.84 (5250, 27.80%)^{b}	9.97±2.73 (5208, 27.83%)	14.38±4 (4823, 27.59%)^{ab}	1.45±0.21 (4823, 27.59%)^{b}	115.08±1.81 (5269, 27.76%)	276.4±48.94 (1293, 29.64%)^{b}	391.63±49.05 (1293, 29.64%)^{b}
Autumn	10.42±2.83 (4494, 23.80%)^{ab}	10.03±2.74 (4444, 23.75%)	14.21±3.94 (4135, 23.65%)^{b}	1.42±0.21 (4135, 23.65%)^{c}	115.14±1.81 (4529, 23.86%)	280.72±50.03 (1028, 23.57%)^{ab}	396.15±50.1 (1028, 23.57%,)^{a}
Winter	10.51±2.99 (4455, 23.59%)^{a}	10.1±2.85 (4421, 23.63%)	14.32±4.02 (4155, 23.76%)^{ab}	1.43±0.21 (4155, 23.76%)^{a}	115.081.85 (4492, 23.66%)	291.42±53.64 (877, 20.11%)^{c}	407.12±53.65 (877, 20.11%)^{c}

TNB, total number born; NBA, number born alive; LBW, litter birth weight; ABW, average birth weight; GL, gestation length; AFS, age at first service; AFF, age at first farrowing.

[1)] The unit of traits are shown in parentheses.

[2)] Phenotypic data of different seasons are displayed by mean±standard deviation;

[3)] The number of individuals and frequencies in different seasons are shown in parentheses, respectively;

[4)] Different superscript letters (a, b, c) in the same column mean significant differences (P<0.05).

Table S5. The effect of parities on reproductive performance traits

Parity	TNB(each) [1)]	NBA(each)	LBW(kg)	ABW(kg)	GL(day)
1	9.96±2.67[2)] (4751, 25.16%)[a]	9.63±2.6 (4661, 24.91%)[3)a]	13.06±3.74 (4304, 24.62%)[a4)]	1.36±0.2 (4304, 24.62%)[a]	115.45±1.88 (4772, 25.14%)[a]
2	10.43±2.9 (3602, 19.08%)[b]	10.07±2.79 (3580, 19.13%)[bc]	14.68±3.93 (3405, 19.47%)[b]	1.47±0.21 (3405, 19.47%)[b]	115.12±1.78 (3627, 19.11%)[b]
3	10.83±2.93 (2909, 15.41%)[de]	10.41±2.82 (2897, 15.48%)[d]	15.2±3.95 (2783, 15.92%)[c]	1.47±0.21 (2783, 15.92%)[b]	114.96±1.75 (2917, 15.37%)[c]
4	11.01±3.03 (2292, 12.14%)[e]	10.5±2.86 (2282, 12.19%)[d]	15.23±4.02 (2188, 12.51%)[c]	1.46±0.21 (2188, 12.51%)[b]	114.87±1.72 (2301, 12.12%)[c]
5	10.85±2.84 (1724, 9.13%)[e]	10.42±2.74 (1712, 9.15%)[d]	14.99±3.83 (1619, 9.26%)[c]	1.45±0.21 (1619, 9.26%)[c]	114.83±1.85 (1736, 9.15%)[c]
6	10.64±2.73 (1303, 6.90%)[cd]	10.17±2.62 (1292, 6.90%)[c]	14.65±3.76 (1157, 6.62%)[b]	1.44±0.2 (1157, 6.62%)[c]	114.86±1.75 (1310, 6.90%)[c]
7	10.46±2.88 (932, 4.94%)[bc]	9.98±2.79 (928, 4.96%)[b]	14.27±3.89 (841, 4.81%)[d]	1.44±0.21 (841, 4.81%)[c]	114.93±1.81 (939, 4.95%)[c]

⩾8	9.88±2.9<br>(1370, 7.26%)[a]	9.39±2.85<br>(1361, 7.27%)[e]	13.18±4<br>(1187, 6.79%)[a]	1.42±0.22<br>(1187, 6.79%)[d]	115.161.75<br>(1380, 7.27%)[b]

TNB, total number born; NBA, number born alive; LBW, litter birth weight; ABW, average birth weight; GL, gestation length; AFS, age at first service; AFF, age at first farrowing.

1) The unit of traits are shown in parentheses.

2) Phenotypic data of different parities are displayed by mean±standard deviation;

3)The number of individuals and frequencies in different parities are shown in parentheses, respectively;

4)Different superscript letters (a, b, c, d, e) in the same column mean significant differences (P<0.05).
